# Meeting of the 22d Annual Meeting of the American Medical Association

**Published:** 1871-09

**Authors:** 


*Minutes of the Twenty-second Annual Meeting of the American Medical Association,* held in the city of San Francisco, May, 1871, pamphlet pp. 43. Price 25 cents.

Owing to some delay, caused in preparing the proceedings of the American Medical Association for the Press, the energetic permanent Secretary, Dr. Wm. B. Atkinson, has published the minutes of the same in a convenient pamphlet form. Those who desire the pamphlet may procure it by inclosing price and addressing the permanent Secretary, Wm. B. Atkinson, M.D., 1400 Pine Street, Philadelphia.

